# Cigarette smoking is associated with levels of the serotonin transporter in the brain: a [^11^C]DASB PET Study

**DOI:** 10.1093/ijnp/pyaf026

**Published:** 2025-04-21

**Authors:** Paul Faulkner, Gitte M Knudsen, Vibe G Frokjaer, David Erritzoe

**Affiliations:** Department of Psychology, Queen Mary University of London, London, United Kingdom; Department of Clinical Medicine, University of Copenhagen, Copenhagen, Denmark; Neurobiology Research Unit, Copenhagen University Hospital Rigshospitalet, Copenhagen, Denmark; Department of Clinical Medicine, University of Copenhagen, Copenhagen, Denmark; Neurobiology Research Unit, Copenhagen University Hospital Rigshospitalet, Copenhagen, Denmark; Psychiatric Center Copenhagen, Copenhagen, Denmark; Centres for Neuropsychopharmacology and Psychedelic Research, Division of Psychiatry, Department of Brain Sciences, Imperial College London, London, United Kingdom

**Keywords:** smoking, nicotine, serotonin, 5-HT, positron emission tomography

## Abstract

**Background:**

Preclinical work suggests that chronic nicotine/tobacco use is associated with reductions in serotonin within the hippocampus, yet no research has yet shown an association of smoking behaviors and alterations in brain serotonin in humans in vivo.

**Methods:**

We therefore analyzed existing [^11^C]DASB PET data from the Cimbi Database to compare the availability of the serotonin transporter (SERT) in the hippocampus, midbrain (including the raphe), and neocortex of 60 healthy non-smokers, 15 ex-smokers, and 11 current smokers who also provided blood samples for determination of plasma tryptophan load. Because SERT availability is considered to be negatively associated with extracellular serotonin levels, we hypothesized that current smokers would exhibit greater SERT availability than ex-smokers and non-smokers.

**Results:**

There was a significant main effect of group on SERT binding (DASB BP_ND_) values in the bilateral and left hippocampus, and a trend toward such in the right hippocampus. Post hoc ANOVAs revealed that current smokers exhibited greater hippocampal DASB BP_ND_ than both non-smokers and ex-smokers, while the latter 2 groups did not differ. There were no group effects on DASB BP_ND_ within the midbrain or global neocortex. Finally, there was no significant group effect on plasma tryptophan load.

**Conclusions:**

This study provides the first in vivo evidence that current smoking may be associated with elevated hippocampal SERT binding—possibly reflecting lower synaptic serotonin concentrations, and that this change may normalize following smoking cessation.

**Significance Statement**

Previous in vivo animal and postmortem human research indicates that nicotine and tobacco use is associated with lower concentrations of serotonin in the hippocampal complex. We provide the first in vivo evidence in humans that cigarette smoking may result in lower concentrations of serotonin within this brain region. Using [^11^C]DASB positron emission tomography, we find that compared to both non-smokers and ex-smokers, current smokers exhibit greater hippocampal availability of the serotonin transporter. We propose that this may reflect that smokers have elevated hippocampal extracellular levels of serotonin which in turn leads to a decrease in the serotonin transporter. If this assumption is correct and since we find no difference between the ex-smokers and non-smokers, we further propose that smoking-related alterations in concentrations of serotonin within the hippocampus normalize following smoking cessation.

## INTRODUCTION

Tobacco use is the leading promoter of disease and death worldwide, contributing to more than 8 million deaths per year around the globe.^1^ Understanding the neurochemical mechanisms of tobacco use disorder could improve public health by aiding the development of novel cessation aids. So far, most research has focused on understanding the association of smoking behaviors and brain dopamine (eg, Brody et al.^2,3^), glutamate,^4,5^ and gamma-aminobutyric acid (GABA),^6^ and often ignores such associations with serotonin (5-HT).

Nevertheless, there is some indication that smoking may have deleterious effects on serotonin function within the hippocampus. For example, in rodents, acute administration of nicotine releases serotonin within the hippocampus,^7^ yet chronic nicotine administration for 20 consecutive days decreases concentrations of serotonin in this brain region.^8^ In humans, the *postmortem* tissue of daily smokers (compared to that of non-smokers) exhibited lower levels of serotonin and higher levels of the inhibitory serotonin 1A (5-HT_1A_) receptor in the hippocampus and median raphe nucleus.^9^ Further, acute smoking increases resting-state functional connectivity between the hippocampus and median raphe nuclei, while chronic daily smoking for ~5 years or more is associated with weaker connectivity between these 2 regions.^10^ Taken together, these results suggest that initially, smoking may increase functioning within brain regions heavily innervated by the serotonin system, but that over time, smoking may decrease functioning within these same regions.

However, the effects of current tobacco use on in vivo markers of serotonin in humans are poorly understood, as are the effects of smoking cessation on such markers. Importantly, while the “smoking” group in the postmortem study conducted by Benwell et al^9^ consisted of 12 individuals who smoked up until death, the “non-smoking” group consisted of 18 individuals, some of whom had given up smoking at least 5 years (and on average 18 years) prior to death. The authors of this study did not report the exact number of such “ex-smokers” in this non-smoking group and did not examine whether non-smokers, smokers, and ex-smokers differed with regard to the above serotonin markers. As such, it is unknown whether ex-smokers display similarly low levels of serotonin as current smokers, or whether smoking cessation is associated with a reversal of these effects on brain serotonin.

The true association between chronic smoking behaviors and serotonin function within the hippocampus in vivo is perhaps best determined using positron emission tomography (PET) to determine the regional availability of the serotonin transporter (SERT). Erritzoe et al^11^ used [^11^C]DASB PET to compare SERT binding between current smokers and non-smokers in 3 main regions of interest (ROIs); a high-binding subcortical region consisting of the caudate, putamen, and thalamus, a midbrain region (including the raphe nuclei) and neocortex, and report no group differences in SERT availability in any of these ROIs. Further, Smolka et al^12^ reported no differences between midbrain SERT binding of a smoking and non-smoking group, though they did show that current smokers with the LL variant of the 5HTTLPR genotype exhibited slightly altered midbrain SERT availability than non-smokers with the same variant, whereas there was no such effect of smoking status on such availability when comparing carriers of an S allele. However, neither Erritzoe et al^11^ nor Smolka et al^12^ examined SERT availability in the hippocampus, while both of these studies compared a group of smokers with a group of non-smokers, the latter of which included ex-smokers. As such, neither study was able to reliably examine whether smoking cessation normalizes SERT availability.

We, therefore, aimed to compare the availability of the SERT in the hippocampus of 3 separate groups of non-smokers, current smokers, and ex-smokers using [^11^C]DASB PET data that is available in the Cimbi database^13^ (https://nru.dk/index.php/research-menu/research-groups/115-the-cimbi-database-and-biobank). As stated above, prior literature indicates that smokers may exhibit lower levels of hippocampal serotonin than non-smokers.^9^ Research indicates that availability of the postsynaptic 5-HT_2A_ receptor in vivo is a surrogate marker of cerebral serotonin levels, with high extracellular serotonin levels being associated with low 5-HT_2A_ receptor binding and vice versa.^14-16^ Importantly, Erritzoe et al^17^ report there to be an inverted U-shaped relationship between 5HT_2A_ receptor availability and SERT binding in the neocortex of healthy individuals, such that low 5-HT_2A_ receptor availability in the neocortex was associated with both low and high SERT availability. Further, research also indicates that elevated extracellular levels of serotonin are associated with low levels of SERT. For example, chronic administration of selective serotonin-reuptake inhibitors (including sertraline, citalopram, and paroxetine) has been shown to result in both elevated levels of extracellular serotonin as well as simultaneous decreased levels of SERT,^18-21^ while monoamine oxidase A knockout mice exhibit increased extracellular serotonin levels and reduced SERT levels.^22^ On the basis of these studies, it may be that high levels of SERT availability in a brain region reflect low levels of serotonin. It was, therefore, hypothesized that, compared to non-smokers and ex-smokers, current chronic smokers would exhibit greater SERT availability in the hippocampus (which would reflect low levels of serotonin in this brain region), and that greater lifetime tobacco use would also be associated with greater hippocampal SERT availability. We also assessed the association of smoking behaviors with DASB BP_ND_ (Non-Displaceable Binding Potential) in the midbrain and global neocortex, for completeness. Finally, to address potential sources for acute effects of smoking on serotonin levels, we examined (post hoc) the influence of group on total plasma tryptophan load, ie, the tryptophan relative to the sum of large neutral amino acids (LNAAs) that are carried across the blood-brain barrier.^23^

## EXPERIMENTAL PROCEDURES

### Participants

Data from 86 participants (60 non-smokers, 15 ex-smokers, 11 current daily smokers) who had undergone [^11^C]DASB PET scanning were included. Non-smokers were defined as such because they reported smoking 0 cigarettes every day without any history of ever smoking; ex-smokers were defined as such because they self-reported a previous history of daily or non-daily smoking; and current smokers were defined as such because they self-reported daily smoking at the time of the scan; importantly, no smokers self-reported non-daily smoking and so none were defined as such. Exclusion criteria were lifetime neurological and psychiatric disorders, substance use disorder (other than tobacco use disorder), left-handedness, pregnancy, and any contraindication for MRI. Written, informed consent was obtained according to the declaration of Helsinki II, and the study was approved by the Copenhagen Region Ethics Committee. All data were collected at the Rigshospitalet in Copenhagen, Denmark, between August 2005 and March 2008. Data were obtained from the Cimbi database and were collected from across 8 studies. The ethical approval numbers of these studies are available on the Cimbi database website (https://nru.dk/index.php/research-menu/research-groups/115-the-cimbi-database-and-biobank), and are as follows: 1. VEK H-1-2010-091 (*N* = 24); 2. VEK H-1-2010-085 (*N* = 9); 3. VEK H-2-2010-108 (*N* = 9); 4. VEK (KF)01-156/04 (*N* = 7); 5. VEK (KF)11-061/03 (*N* = 9); 6. VEK (KF)02-058/99 (*N* = 7); 7. VEK KF-01-124/04 (*N* = 18); and 8. VEK (KF)01-2006-20 (*N* = 3). None of the PET scans included in the current dataset were conducted after the administration of any intervention. Importantly, participants were either scanned using a Siemens high-resolution research tomograph (HRRT) PET scanner (studies 1, 2, and 3; total *N* = 42, consisting of 34 non-smokers, 6 ex-smokers, 2 current smokers) or a GE Advance PET scanner (studies 4, 5, 6, 7, and 8; total *N* = 44, consisting of 26 non-smokers, 9 ex-smokers, 9 current smokers). All our analyses controlled for the effect of scanner type.

### Questionnaire Measures

Daily cigarette use was quantified as the average number of cigarettes smoked per day, as determined using a questionnaire developed in-house. Lifetime tobacco use was inferred from “pack years,” calculated as the average number of cigarette packets smoked per day multiplied by the number of years of smoking, as in previous studies.^5,24,25^ Because smoking may negatively influence mood,^25^ and because the brain’s serotonin system is implicated in experiencing symptoms of depression and anxiety, we used the 12-item Major Depression Index (MDI)^26^ to quantify depression (with a score of 0-19 indicating no depression), and the 90-item Symptom Checklist-90 (SCL-90)^27^ to quantify depression and anxiety (with a score of <1.6 and <1.15 representing no depression and no anxiety, respectively).

### PET Imaging Procedure

The PET imaging procedure was identical for both scanner types used and can be seen in detail in Erritzoe et al,^11^ Nørgaard et al,^28^ Knudsen et al,^13^ and the Supplementary Materials. Briefly, scans were acquired using either an 18-ring GE Advance scanner (General Electric), or a HRRT PET scanner (CTI/Siemens). After a 10-minute transmission scan, a dynamic 90-minute recording was initiated upon intravenous injection, which consisted of 36 frames (35 3D image slices per frame, interslice interval of 4.25mm), each of which increased in duration from 10 seconds to 10 minutes. The attenuation-corrected and decay-corrected recordings were reconstructed by filtered-back projection using a 6 mm Hann filter.

### MR Imaging Procedure

The magnetic resonance imaging (MRI) imaging procedure can also be seen in detail in Erritzoe et al,^11^ Nørgaard et al,^28^ and Knudsen et al.^13^ Briefly, 3D T1-weighted sagittal magnetization-prepared rapid gradient echo (MPRAGE) scans of the head (echo time  = 3.93 ms; repetition time  = 1540 ms; inversion time  = 800 ms; slice resolution = 75%; bandwidth = 130 Hz/Px; echo spacing = 9.8 ms) were acquired using a Siemens Magnetom Trio 3T scanner with an 8-channel head coil (In vivo).

To enable extraction of the PET voxel of interest-signal from voxels within gray matter only, MPRAGE scans were segmented into gray matter, white matter, and cerebrospinal fluid tissue using SPM2. This was done for the neocortex and the hippocampus, but not for the midbrain as segmentation within this region is considered unreliable; all midbrain voxels were included in analyses involving this region.

### Movement Correction and Coregistration of PET to MR Images

All time frames of the attenuation-corrected emission recording were automatically aligned to a mid-scan frame (ie, frame 26) using the Automate Image Registration (AIR) algorithm (https://www.nitrc.org/projects/air/). We then calculated the mean PET image for frames 10-36 for coregistration to the individual MR image using this AIR algorithm (coregistration quality was controlled visually). Partial volume correction was performed as described in Erritzoe et al^11^ and Nørgaard et al.^28^

### Volume of Interest Analysis

Regions of interest were automatically delineated on each subject’s T1-weighted MR image using the PVElab software package (https://nru.dk/index.php/allcategories/download/37-pvelab/131-pvelab),^29^ as described in detail in both Erritzoe et al^11^ and in the Supplementary Materials.

Volume-weighted SERT binding values were calculated from the hippocampus. We also examined SERT availability in the global neocortex and in the midbrain (including the raphe) for completeness; these results can be seen in the Supplementary Materials. The global neocortex region consisted of a volume-weighted average of the orbitofrontal, medial inferior frontal, superior frontal, superior temporal, medial inferior temporal, sensory motor, parietal, and occipital cortices. The delineation of all ROIs has been described previously,^29^ except for the midbrain which was defined in the plane of the anterior and posterior commissure as the superior limit and the border between the inferior colliculi and the superior cerebellar peduncle as the inferior limit; in the 2-3 most superior slices where the peduncle is less well-defined, only the tegmentum and tectum were included.

### Quantification of BP_ND_

The outcome parameter from the [^11^C]DASB PET data is BP_ND_. The cerebellum was used as a reference region, representing non-displaceable uptake only. We quantified [^11^C]DASB BP_ND_ using a modified reference tissue model (MRTM/MRTM2) designed specifically for the quantification of this ligand,^30^ using PMOD (v2.9). A fixed washout constant (k2′) was calculated for each scan as an average k2 in brain regions relative to the reference region (cerebellum, representing nonspecific binding) using MRTM. Subsequently, k2′ was inserted into MRTM2, and BP_ND_ was calculated, weighting these values by the gray matter volume of the relevant brain region.

#### Plasma Tryptophan Load—

Because it may be that high levels of tryptophan (ie, the precursor to serotonin) in the brain could alter hippocampal SERT availability by influencing brain levels of serotonin, we collected blood samples to calculate tryptophan load (ie, the concentration of tryptophan relative to its other carrier-competing amino acids) in the plasma. Importantly, we calculated this variable, as opposed to simply calculating plasma concentrations of tryptophan alone, because tryptophan crosses the blood-brain barrier via competition with other LNAAs at a common transport mechanism, meaning that levels of plasma tryptophan load (as defined above) are thought to give a more accurate depiction of levels of tryptophan in the brain than concentrations of tryptophan on its own.^23^ Blood samples were collected in heparinized vials, centrifuged, and stored at −80°C until analyzed. For analysis, sulfosalicylic acid was added to precipitate the protein, and norleucine was added as an internal standard. Large neutral amino acids were measured in the plasma with high-pressure liquid chromatography, and the tryptophan load (as defined above) was calculated.

#### Data Analyses—

The effect of the group on BP_ND_ and plasma tryptophan values was determined using ANOVAs that contained the relevant scores as the dependent variable, and group (current smokers vs ex-smokers vs non-smokers) as a categorical factor.

Relationships between both DASB BP_ND_ and plasma tryptophan levels with continuous measures of smoking behaviors (mean cigarettes smoked per day in smokers only, and pack years in both the current and ex-smokers combined) were calculated using correlational analyses.

Because DASB BP_ND_ can be influenced by age,^31^ sex,^32^ body mass index (BMI),^11^ and the number of daylight minutes on the day of scanning,^33^ all analyses that examined the effects of smoking behaviors (ie, smoking status and pack years/cigarettes per day) on DASB BP_ND_ and plasma tryptophan values controlled for the effects of these variables. Body mass index was calculated by recording the weight and height of each participant, while the number of daylight minutes on the day of PET scanning was calculated based on data at https://aa.usno.navy.mil/data/Dur_OneYear. In addition, because DASB BP_ND_ levels differed as a function of the scanner type used (see results below and Table S1), all analyses also controlled for the effect of scanner type on the relevant dependent variable. Specifically, this was by including scanner type as a categorical covariate of no interest in the model, which in turn statistically adjusted the relevant dependent variable based on the influence of scanner type, essentially removing its confounding impact on the relationship between the independent variable of interest (eg, group) on the dependent variable, as was performed by the previous studies that examined the relationship between nicotine/drug use and neurochemistry using PET data collected from different scanners^34,35^ and by the CIMBI group when using PET data to examine the role of brain serotonin in psychiatry.^36^ All data were normally distributed with the exception of pack years; this variable was rendered normally distributed via a square root transformation. A power analysis detailing the achieved power of our approach can be observed in the Supplementary Materials.

Corrections for multiple comparisons were performed as follows: When performing each analysis, if the relevant omnibus test (which always contained BP_ND_ values in the bilateral hippocampus as the dependent variable) produced a significant result, a Fisher’s least significant difference (LSD) method was used to then perform exploratory analyses of BP_ND_ values in the left and right hippocampus. The primary hypotheses pertained to the hippocampus; the midbrain and neocortex ROIs were examined for completeness.

## RESULTS

### Participant Characteristics

Current smokers self-reported smoking an average of 11.9 (SD = 7.0) cigarettes per day. There was no difference in terms of pack years between ex-smokers (mean = 15.9, SD = 20.9) and current smokers (mean = 5.0, SD = 4.3) (*t*(21) = 1.69, *P* = .11, *2-tailed*) (Table 1). Data pertaining to the amount of time since participants smoked their last cigarette prior to the PET scan were available from only 6 current smokers (mean time since last cigarette = 5.1 [SD = 5.1] *hours* before their PET scan) and only 3 ex-smokers (mean time since last cigarette = 16.8 [SD = 20.1] *years* before their PET scan).

**Table 1.** Participant characteristics.

	Whole group	Non-smokers	Ex-smokers	Smokers	*Group comparison*
*N*	86	60	15	11	-
Sex (*M/F*)	56/30	39/21	10/5	7/4	*P* = .99
Age (*years*) ^a^	30.2 ± 14.6	28.4 ± 12.3	41.0 ± 21.8	25.5 ± 5.6	*P* = .01*
BMI ^a^	24.5 ± 4.2	23.8 ± 3.1	24.7 ± 2.6	28.1 ± 8.2	*P* = .27
Cigarettes per day ^a^	11.9 ± 7.0	-	-	11.9 ± 7.0	-
Pack years ^a^	11.2 ± 16.6	-	15.9 ± 20.9	5.0 ± 4.3	*P* = .11
Daylight minutes ^a^	647.5 ± 251.4	620.0 ± 243.7	657.5 ± 258.6	783.6 ± 260.5	*P* = .14

The red asterisk denotes significant group effect.

^a^Denotes mean and SD.

There was a main effect of group on age (*F*(2,83) = 5.73, *P* = .01), which was driven by ex-smokers being significantly older than both the non-smokers and current smokers (both *p*s < .03). Age was positively associated with pack years in both current smokers and ex-smokers (both *p*s < .01).

There was no difference between groups in the ratio of males to females, BMI, or daylight minutes (all *p*s > .27). In smokers and ex-smokers, sex, BMI, and daylight minutes were not associated with the number of cigarettes smoked per day or pack years (all *p*s > .10).

Participants reported mean scores of 4.05 (SD = 3.09, range = 0-11) on the MDI. In addition, they self-reported mean scores of 0.19 (SD = 0.26, range = 0-0.84) on the depression scale and 0.11 (SD = 0.18, range = 0–0.80) on the anxiety scale of the SCL-90. These results reveal that no participant self-reported symptoms of depression or anxiety.

### Comparison of Siemens HRRT Data and GE Advance Data

In the whole group, there was a main effect of scanner type on DASB BP_ND_ values within the bilateral, left and right hippocampal ROIs, with BP_ND_ being higher in individuals scanned with the HRRT scanner (bilateral hippocampus: *t*(84) = −4.324, *P* < .001; left hippocampus: *t*(84) = 4.46, *P* < .001; right hippocampus: *t*(84) = 3.71, < 0.001) (Table S1). For a full list of participant characteristics by scanner type, see Table S1 in the Supplementary Materials.

### Association of Smoking Behaviors and [^11^C]DASB BP_ND_

**Figure 1.**
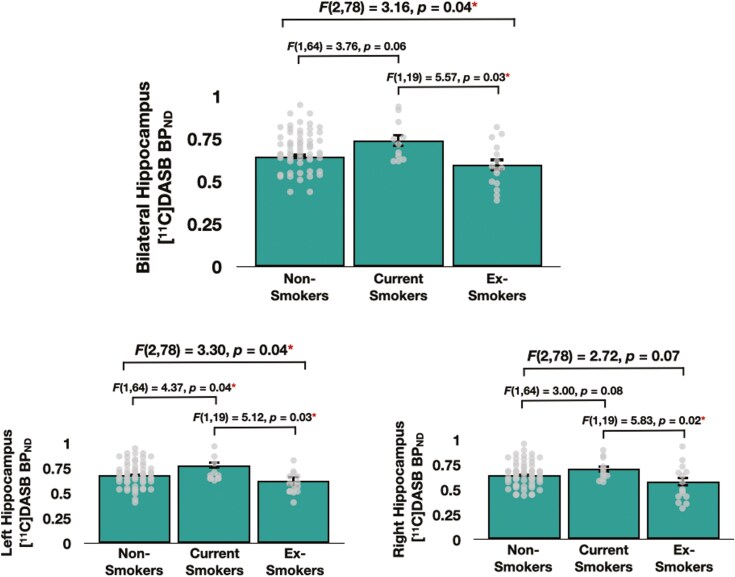
DASB BP_ND_ in the bilateral hippocampus (top), left hippocampus (bottom left), and right hippocampus (bottom right) in non-smokers, current smokers, and ex-smokers. Red asterisks indicate a significant group effect on DASB BP_ND_ values. Dots denote individual data points.

The omnibus ANOVA that controlled for age, sex, BMI, daylight minutes, and scanner type revealed a significant main effect of group on [^11^C]DASB BP_ND_ in the bilateral (*F*(2, 78) = 3.16, *P* = .04) and left hippocampus (*F*(2,78) = 3.30, *P* = .04), while the group effect on DASB BP_ND_ in the right hippocampus just missed the threshold for significance (*F*(2,78) = 2.72, *P* = .07) (Figure 1).

Post hoc ANOVAs that controlled for the same variables revealed that current smokers exhibited *greater* [^11^C]DASB BP_ND_ values than ex-smokers in the bilateral hippocampus (*F*(1,19) = 5.57, *P* = .03), left hippocampus (*F*(1,19) = 5.12, *P* = .03), and right hippocampus (*F*(1,19) = 5.83, *P* = .02). Further, current smokers also exhibited *greater* DASB BP_ND_ values than non-smokers in the left hippocampus (*F*(1,64) = 4.37, *P* = .04), but that such group differences in DASB BP_ND_ within the bilateral and right hippocampal ROIs followed a similar trend while narrowly missing significance (bilateral hippocampus: *F*(1,64) = 3.76, *P* = .06; right hippocampus: *F*(1,64) = 3.00, *P* = .08). Finally, non-smokers and ex-smokers did not differ in terms of DASB BP_ND_ values in any hippocampal ROI (all *p*s > .18). There were no significant group effects on BP_ND_ values in the midbrain or global neocortex (both *p*s > .53; see Figure S1).

When controlling for age, sex, BMI, daylight minutes, and scanner type, there were no significant relationships between hippocampal DASB BP_ND_ values and either pack years or the average number of cigarettes smoked per day (all *p*s > .458). For the relationship between DASBP BP_ND_ and time since last smoked, see Figure S2 in the Supplementary Materials.

### Association of Smoking Behaviors and Plasma Tryptophan Load

**Figure 2.**
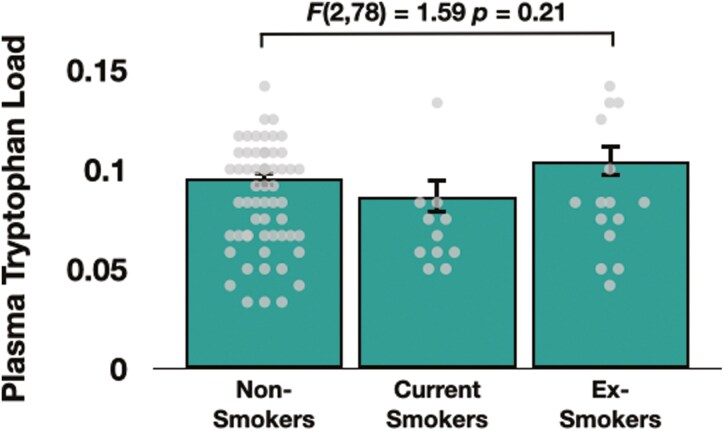
Plasma tryptophan load in non-smokers, current smokers, and ex-smokers. Dots denote individual data points.

The omnibus ANOVA that controlled for age, sex, BMI, daylight minutes, and scanner type revealed no main effect of group on plasma tryptophan load (*F*(2, 78) = 1.59, *P* = .21) (Figure 2). Further, when controlling for these variables, there were no significant relationships between plasma tryptophan load and either cigarettes per day or pack years (both *p*s > .38). Finally, there was a significant relationship between plasma tryptophan load and levels of DASB BP_ND_ in the bilateral hippocampus (*r* = 0.31, *P* = .01), although this relationship was not significant when individuals examining the ex-smoker group (*r* = 0.49, *P* = .07) and non-smoker group (*r* = 0.42, *P* = .19) separately (see Supplementary Materials).

## DISCUSSION

This study shows an association between cigarette smoking and indirect markers of brain serotonin in vivo using PET. Specifically, using [^11^C]DASB PET, this study is the first to reveal that compared to ex-smokers and non-smokers, current daily smokers exhibit greater hippocampal SERT binding.

Our primary hypothesis was that cigarette smoking behaviors are associated with decreased serotonin levels. This was made on the basis of evidence which indicates that current smokers exhibit lower concentrations of serotonin in the hippocampus *post mortem* than non-smokers.^9^ Due to evidence suggesting that low concentrations of serotonin are considered to be associated with high SERT availability in this region,^17-21^ we predicted that cigarette smoking behaviors would be associated with greater SERT availability in the hippocampus; this prediction was indeed supported by the data. It may be that acute smoking-induced increases in hippocampal serotonin observed by Kenny et al,^7^ and as hypothesized by Faulkner et al,^10^ may promote an adaptive upregulation of SERT in this region, as suggested by Wurtman and Wurtman.^37^

Lower hippocampal serotonin due to chronic smoking over many years observed in Benwell et al^9^ (and suggested by the current results) could also partly be caused by smoking-related increases in concentrations of inhibitory 5-HT_1A_ receptors in the hippocampus that was observed in smokers *postmortem*,^9^ or by possible effects on serotonergic projections to the hippocampus. Unfortunately, it is impossible to find support for such a hypothesis from the current study due to the cross-sectional design employed. Future studies should use a longitudinal design to examine the association of smoking behaviors with the availability of the SERT and various serotonin receptors over the lifespan.

Intriguingly, the ex-smokers in this study exhibited very similar DASB BP_ND_ values as the non-smokers. This group had not smoked for ~17 years, indicating that the high DASB BP_ND_ values observed in current smokers may normalize following cessation from smoking. The possibility that such a normalization may occur following smoking cessation could be inferred by the fact that hippocampal DASB BP_ND_ was negatively associated with the number of minutes since last smoking (see Supplementary Materials), as this result indicates that participants who had not smoked for the longest period of time exhibited the lowest SERT availability in this brain region. As such, future studies may wish to examine SERT availability in a larger sample of ex-smokers to better determine whether cessation does indeed “normalize” the availability of the SERT, or whether this effect is due in part to personality characteristics such as age (which has been shown to be associated with SERT availability).^31^

Our analyses revealed no group effect on plasma tryptophan load. Because this was calculated as the concentration of tryptophan relative to its other carrier-competing amino acids, and because this amino acid competes for entry into the brain via the same mechanisms as the other LNAAs, this variable is considered to be a better indication of levels of tryptophan in the brain than concentrations of tryptophan in the plasma.^23^ The present results, therefore, indicate that tobacco smoking does not alter concentrations of tryptophan in the human brain. To date, there have been no other studies that have examined the effects of smoking or nicotine administration on tryptophan load, though studies of the effects of nicotine administration on plasma tryptophan concentrations do provide similar results. For example, Gaynor and Handley^38^ revealed no effect of nicotine administration on plasma concentrations of tryptophan, while Takada et al^39^ report that acute administration of nicotine did not alter concentrations of tryptophan in the plasma. In addition, it is possible that higher SERT availability observed in the current smokers may be partly explained by the lower plasma tryptophan load values observable in Figure 2, as plasma tryptophan load levels were significantly correlated with SERT availability in the whole group, and moderately, albeit non-significantly, in the current smokers group.

This study has some limitations. First, the current smokers self-reported a relatively low level of lifetime tobacco use, and assessing smokers with a wide range of lifetime tobacco use would have provided more detailed information regarding the effects of cigarette use on brain serotonin. Second, a clear picture of brain serotonin function is very difficult to obtain by quantifying the in vivo binding of just one aspect of the serotonin system. As such, future studies may wish to determine the association of smoking behaviors with the availability of the SERT as well as with a range of serotonin receptors, or with dynamic changes in serotonin release capacity.^40,41^ Third, examining SERT availability within a region that contains all structures within the midbrain does not allow for a definitive examination of SERT availability within the raphe nuclei. While this brain region has proved difficult to delineate in the past, studies have been able to successfully do so by using a mask of the raphe first defined in Beliveau et al^42^ (eg, Faulkner et al.^10^). Fourth, while our sample size provided adequate statistical power to detect large and medium effects (93%+ and 85%+ power) in the data, it provided very low power to detect smaller effects (<60% power), meaning that we were unable to examine the role of individual differences in the data. As such, future studies may wish to examine the effects of smoking behaviors on brain serotonin in a larger sample of smokers. This may be particularly important when trying to examine the effects of individual differences in factors that can affect SERT availability. For example, Erritzoe et al^11^ reported that BMI is negatively associated with SERT availability, particularly in individuals with BMI > 30 (defined as obesity). Despite the fact that BMI did not differ by group in the current study, 27% of our current smokers (3/11) had a BMI > 30 compared to 3% (2/60) of our non-smokers and 0% of our ex-smokers, there is a slight possibility that this variable may have influenced our findings. In addition, the fact that there are a smaller number of females than males in each group inhibited our ability to determine the role of sex/gender differences in the effects of smoking on brain serotonin function. Importantly, research indicates that compared to men, women may exhibit lower SERT availability in many brain regions including the hippocampus.^32^ Furthermore, SERT availability decreases with increasing age, and these age-related changes may vary from brain region to region.^43^ Because the mean age was greater in the ex-smokers than in the other groups, future studies may also wish to examine the effects of smoking on brain serotonin in a sufficiently large sample to enable reliable examination of the role of individual differences in age, gender, and BMI. Furthermore, this small sample size may influence the reliability of a Fisher’s LSD for multiple comparisons due to the dependence of this approach on the initial omnibus test. Finally, because hippocampal BPND values were higher in those individuals who were scanned using the HRRT PET scanner, and because a smaller proportion of the current smokers were scanned with this HRRT scanner (ie, 2 vs 9 who were scanned using the GE Advanced PET scanner), it is entirely possible that the differences in SERT binding between the current-smokers vs non-smokers and/or ex-smokers are in reality larger than reported here. Future studies may therefore wish to compare SERT availability in a large sample of current, ex-, and non-smokers using only one PET scanner type. Finally, the cross-sectional nature of this study design limited our ability to determine the causal effects of smoking (and indeed other variables such as age, gender, and BMI) on SERT availability. As such, it would be useful to undertake a longitudinal examination of the effects of smoking on brain serotonin to adequately determine the causal effects of tobacco use on the functioning of this neurotransmitter system.

In conclusion, this study provides the first in vivo evidence in humans that cigarette smoking may be associated with a reduction in serotonin within the hippocampus, and that this may normalize following smoking cessation. Future research is needed to achieve a more complete understanding of the effects of smoking on other aspects of serotonergic architecture and to determine the ability of smoking to acutely alter serotonin function.

## Supplementary Material

pyaf026_suppl_Supplementary_Materials

## Data Availability

Data are held within the CIMBI dataset and are available upon request.
