# Supplementary material for: Cigarette smoking is associated with levels of the serotonin transporter in the brain: a [11C]DASB PET Study
Source: Int J Neuropsychopharmacol. 2025 Apr 21;28(5):pyaf026. doi: 10.1093/ijnp/pyaf026 (PMC12095803; doi:10.1093/ijnp/pyaf026)
Supplement: pyaf026_suppl_Supplementary_Materials [file pyaf026_suppl_supplementary_materials.docx]

**Supplementary Materials**

**Supplementary Methods**

***PET Imaging Procedure***

PET scans were performed using an 18-ring GE-Advance scanner (General Electric, Milwaukee, Wisconsin), which operated in three-dimensional acquisition mode and produced 35 image slices per scan, with an interslice interval of 4.25mm. After a 10-minute transmission scan, a dynamic 90-minute emission recording was initiated upon intravenous injection during 12 seconds of mean 487 (SD = 90) MBq (range = 246 – 601) [^11^C]DASB with a mean specific activity of 32 (SD = 12) GBq/μmol (range = 9 – 82). The emission recording consisted 36 frames, each of which increased progressively in duration from 10 seconds to 10 minutes. The attenuation-corrected and decay-corrected recordings were reconstructed by filtered-back projection using a 6mm Hann filter.

***MR Imaging Procedure***

Structural brain scans were acquired on a Siemens Magnetom Trio 3T scanner with an eight-channel head coil (In vivo, Florida, USA). All participants underwent a high-resolution 3D T1-weighted sagittal magnetization-prepared rapid gradient echo (MPRAGE) scan of the head (echo time (TE)=3.93msec; repetition time (TR)=1540msec; inversion time (TI)=800msec; slice resolution=75%; bandwidth=130 Hz/Px; echo spacing=9.8msec).

To enable extraction of the PET voxel of interest (VOI)-signal from voxels within gray matter only, T1-weighted MR images were segmented into gray matter, white matter and cerebrospinal fluid tissue classes using SPM2 (<https://www.fil.ion.ucl.ac.uk/spm/software/spm2/>) and the Hidden Markov Random Field (HMRF) model as implanted in the SPM2 VBM toolbox (<http://dbm.neuro.uni-jena.de/vbm/>). This was done for the subcortical high-binding regions and for the neocortex, but not for the midbrain as segmentation within this region is considered unreliable; all midbrain voxels were included in analyses involving this region.

***Movement Correction and Co-Registration of PET to MR Images***

All time frames of the attenuation-corrected emission recording were automatically aligned to frame 26 using the Automate Image Registration (AIR) algorithm (<https://bishopw.loni.ucla.edu/AIR5/>). In the next step, we calculated the mean PET image for frames 10-36 for coregistration yo the individual MR image, again using the AIR algorithm. The quality of each coregistration was controlled visually. Partial volume correction was performed as previously described in detail (Erritzoe et al., 2010; Kalbitzer et al., 2009).

***Volume of Interest Analysis***

Regions of interest (ROIs) were automatically delineated on each subject’s T1-weighted MR image in a user-independent fashion with the Pvelab software package ([www.nru.dk/downloads](http://www.nru.dk/downloads)) (Svarer et al., 2005). A 12-parameter affine transformation and a warping field were calculated between the template MR image and each individual participant’s T1-weighted MR image. Having obtained the transformation parameters from the MR/PET coregistration step described above, the template ROIs were then transferred to the dynamic PET image space for each participant. Gray matter time activity curves (TAC) were then extracted from these ROIs. The TAC extracted from the cerebellum, excluding the cerebellar vermis (Kish et al., 2005) was used as the reference tissue input for kinetic modelling.

Volume-weighted SERT binding values were calculated from the hippocampus, as well as from the midbrain. The global neocortex region consisted of a volume-weighted average of the orbitofrontal, medial inferior frontal, superior frontal, superior temporal, medial inferior temporal, sensory motor, parietal and occipital cortices. The delineation of all ROIs has been described previously (Svarer et al., 2005), except for the midbrain which was defined in the plane of the anterior and posterior commissure as the superior limit and the border between the inferior colliculi and the superior cerebellar peduncle as the inferior limit. In the 2-3 most superior slices where the peduncle is less well-defined, only the tegmentum and tectum were included in the region.

***Quantification of Non-Displaceable Tracer Uptake***

The outcome parameter from the [^11^C]DASB-PET data is the non-displaceable binding potential (BP_ND_). The cerebellum was used as a reference region, representing non-specific binding only. We used a modified reference tissue model (MRTM/MRTM2) designed specifically for quantification of this ligand, as described in Ichise et al (2003), using the software PMOD (v2.9); a fixed washout constant (k2’) was calculated for each scan as an average k2 in the relevant brain regions relative to the cerebellum using MRTM. Subsequently, k2’ was inserted into MRTM2 and BP_ND_ was calculated.

***Data Analysis – Power Analysis***

This sample size of 86 (60 non-smokers, 15 ex-smokers, 11 current daily smokers) provided 93%, 80% and 57% power to detect a large (Cohen’s *f* > 0.40), medium (Cohen’s *f* = 0.25 – 0.39) and small (Cohen’s *f* = 0.10 – 0.24) effect of group on DASB BP_ND_ (when controlling for the 5 variables of age, sex, BMI, number of daylight minutes and scanner type). Further, the sample size of 15 ex-smokers and 11 current daily smokers provided 97%, 85% and 44% power to detect large (*r* > 0.80), medium (*r* = 0.31 – 0.50) and small (*r* > 0.30) correlations between smoking behaviours (i.e. pack years) and plasma tryptophan load.

**Supplementary Results**

***Association of Scanner Type and Participant Characteristics***

There was no association between scanner type (GE Advance vs Siemens HRRT) on group composition (i.e. the ratio of current- vs ex- vs non-smokers), cigarettes smoked per day, pack years or daylight minutes (all *p*s > 0.104). However, compared to those who underwent scanning on the Siemens HRRT scanner, participants who were scanned on the GE Advance scanner were significantly older (*t*(84) = 3.499, *p* < 0.001) and had a greater mean BMI (*t*(84) = 3.161, *p* = 0.001). For participants characteristics for each scanner type, see Table S1.

***Association of Time Since Last Smoked and [^11^C]DASB BP_ND_***

Bivariate correlational analyses revealed that in the 11 participants (8 current smokers and 3 ex-smokers) who provided data pertaining to the number of minutes since they last smoked their cigarette (i.e. prior to their PET scan), there was a significant negative correlation between the number of minutes since the last cigarette and DASB BP_ND_ in the bilateral hippocampus (*r* = -0.68, *p* = 0.04) and left hippocampus (*r* = -0.74, *p* = 0.02), and a trend towards such in the right hippocampus (*r* = -0.600, *p* = 0.090).

When splitting these 9 participants into the 6 current smokers and 3 ex-smokers, there were no such significant associations despite the relationships being moderate to strong, likely due to the very small sample size. Specifically, in the 3 ex-smokers, there were non-significant negative associations between the number of minutes since the last cigarette and DASB BP_ND_ in the bilateral hippocampus (*r* = -0.944, *p* = 0.214), left hippocampus (*r* = -0.871, *p* = 0.327), and a significant association between time since last smoked and DASB BP_ND_ in the right hippocampus (*r* = -0.999, *p* = 0.004). Finally, in the 6 current smokers, there were non-significant negative associations between the number of minutes since the last cigarette and DASB BP_ND_ in the bilateral hippocampus (*r* = -0.30, *p* = 0.476), left hippocampus (*r* = -0.39, *p* = 0.341), and right hippocampus (*r* = -0.15, *p* = 0.721) (see Figure S2).

**Supplementary Figure**

***
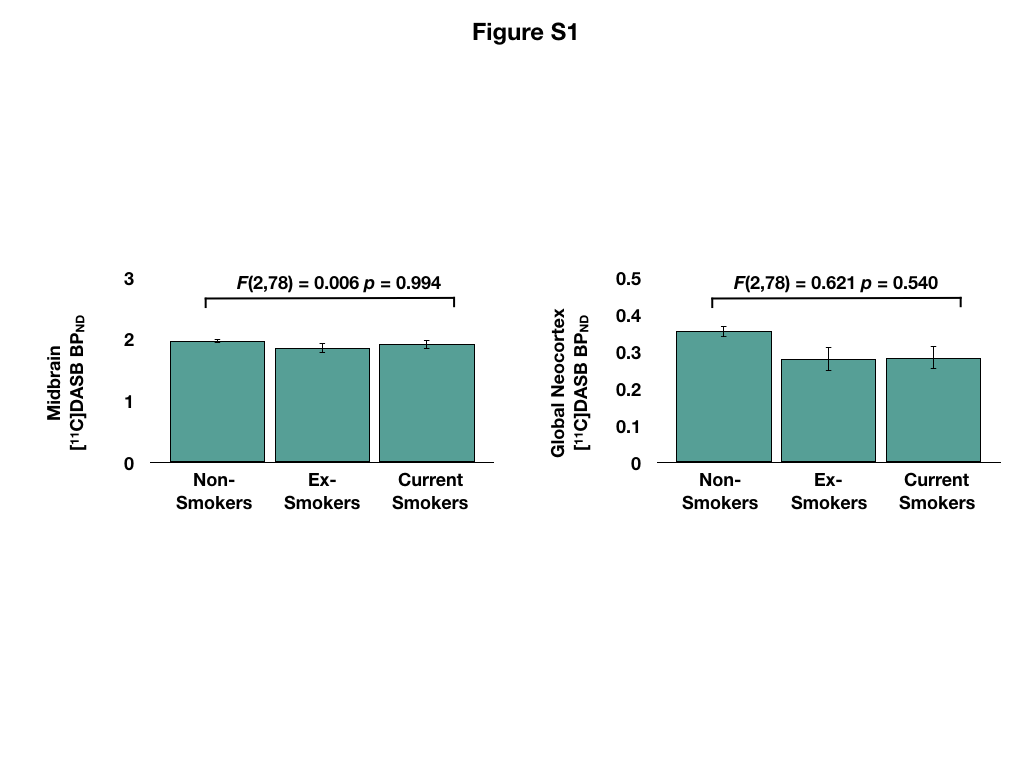
***

***Figure S1:*** SERT availability in the midbrain (left) and global neocortex in current smokers, ex-smokers and non-smokers. Statistics denote no significant group effect on DASB BP_ND_ in either of these brain regions

**
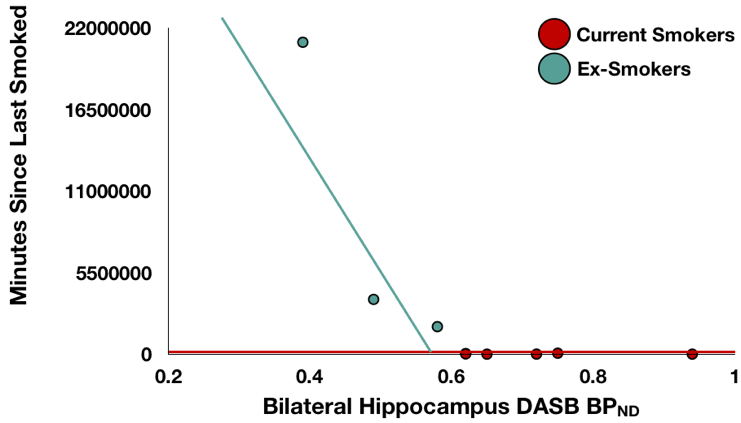
**

***Figure S2:*** Association between Minutes Since Last Smoked and SERT availability in bilateral hippocampus in current smokers (red) and ex-smokers (green).

Finally, there was a significant relationship between plasma tryptophan load and levels of DASB BP_ND_ in the bilateral hippocampus (*r* = 0.305, *p* = 0.01), although this relationship was not significant when individual examining the ex-smoker group (*r* = 0.485, *p* = 0.07) and non-smoker group (*r* = 0.420, *p* = 0.19).

***Association of Smoking Behaviours and Plasma Tryptophan Load (Figure 2)***

There was a significant relationship between plasma tryptophan load and levels of DASB BP_ND_ in the bilateral hippocampus (*r* = 0.30, *p* = 0.01), left hippocampus (*r* = 0.32, *p* = 0.01) and right hippocampus (*r* = 0.25, *p* = 0.02). When examining each group separately, the relationships were significant in the non-smoking group (plasma tryptophan load and bilateral hippocampus: *r* = 0.31, *p* = 0.02; left hippocampus: *r* = 0.31, *p* = 0.02; right hippocampus: *r* = 0.29, *p* = 0.02). In the ex-smoking group, plasma tryptophan load significantly correlated with DASB BPND in the left hippocampus (*r* = 0.53, *p* = 0.04), but not in the bilateral hippocampus (*r* = 0.49, *p* = 0.07) or right hippocampus (*r* = 0.35, *p* = 0.19). Finally, in the current-smoking group, plasma tryptophan load did not correlate with DASB BPND values in any of the bilateral hippocampus (*r* = 0.42, *p* = 0.20), left hippocampus (*r* = 0.52, *p* = 0.10) or right hippocampus (*r* = 0.30, *p* = 0.36).

**Supplementary Table**

|  | **Whole Group** | **GE Advance** | **HRRT** | ***Comparison*** |
| --- | --- | --- | --- | --- |
| *N* | 86 | 44 | 42 | - |
| *Males/Females* | 56/30 | 28/16 | 28/14 | *p* = 0.961 |
| *Non-Smokers/Ex-Smokers/Current Smokers* | 60/15/11 | 26/9/9 | 34/6/2 | *p* = 0.782 |
| Age (*years*) ***^a^*** | 30.23 ± 14.59 | 35.20 ± 2.77 | 25.03 ± 0.88 | *p* = 0.006 ***** |
| BMI  ***^a^*** | 24.53 ± 4.19 | 25.84 ± 0.75 | 23.15 ± 0.39 | *p* = 0.017 ***** |
| Cigarettes Per Day ***^a^*** | 11.90 ± 6.97 | 11.75 ± 2.41 | 12.50 ± 7.50 | *P* = 0.901 |
| Pack Years ***^a^*** | 11.17 ± 16.63 | 10.32 ± 3.56 | 8.07 ± 3.83 | *p* = 0.105 |
| Daylight Minutes *^a^* | 647.47 ± 251.35 | 690.32 ± 37.35 | 602.57 ± 38.60 | *p* = 0.106 |

**Table S1.** Participant Characteristics. Red asterisks denote significant group effect. *^a^* denotes mean and standard deviation.
